# Retraction: Impact of the hydraulic fracturing on indoor radon concentrations in Ohio: a multilevel modeling approach

**DOI:** 10.3389/fpubh.2025.1604930

**Published:** 2025-04-08

**Authors:** Frontiers Editorial Office

The journal retracts the 10th April 2019 article cited above.

Following publication, the authors contacted the Editorial Office to request the retraction of the cited article, stating that it was found after publication that the input file may not be correct and that the fracking well data set used to correlate with the indoor radon data requires additional data validation. Therefore, the findings reported in the article are no longer supported by the analyses. An investigation was conducted in accordance with Frontiers' policies that confirmed this; therefore, the article has been retracted. The authors sincerely apologise for this error and any inconvenience caused.

